# Psychological predictors of future dangerous behavior among probationers: evidence from judicial bureaus in four provinces in China

**DOI:** 10.3389/fpsyg.2025.1593698

**Published:** 2025-10-29

**Authors:** Ling Tang, Tun Xu, Junming Ma

**Affiliations:** ^1^School of Law, China Jiliang University, Hangzhou, China; ^2^Mental Health Center, Shandong Urban Construction Vocational College, Jinan, China

**Keywords:** future dangerous behaviors, relative deprivation, negative coping styles, self-esteem, community corrections, judicial bureaus

## Abstract

**Introduction:**

The psychological mechanisms of future dangerous behaviors among probationers may be influenced by multiple factors. This study aims to explore the impact of relative deprivation on probationers’ future dangerous behaviors, with a focus on the mediating role of negative coping styles and the moderating effect of self-esteem on this pathway.

**Methods:**

A survey was conducted among 1,023 probationers from 48 judicial offices across four provinces in China. Data were collected using the Future Dangerous Behaviors Questionnaire (FDQ), Relative Deprivation Questionnaire (RDQ), Coping Style Questionnaire (CSQ), and Self-Esteem Scale (SES). The moderated mediation model was tested using SPSS PROCESS macro 3.3 software, with negative coping styles as the mediator and self-esteem as the moderator, while controlling for age, gender, education, crime type, sentencing term, and residency status.

**Results:**

Relative deprivation was a significant predictor of probationers’ future dangerous behaviors. Negative coping styles partially mediated the relationship between relative deprivation and future dangerous behaviors, while self-esteem negatively moderated both the direct effect of relative deprivation on future dangerous behaviors and the mediating effect of negative coping styles.

**Conclusion:**

This study demonstrates the pivotal roles of subjective perception (relative deprivation), psychological mechanisms (negative coping styles), and personal resources (self-esteem) in shaping probationers’ future dangerous behaviors. Therefore, judicial officials should integrate community-based strategies to enhance probationers’ adaptive coping skills and improve their self-esteem, thereby reducing the likelihood of further dangerous behaviors and promoting successful reintegration into society.

## 1 Introduction

Probation plays an irreplaceable role in compensating for the shortcomings of imprisonment and implementing criminal policies. According to Articles 72 and 76 of China’s Criminal Law and Article 269 of the Criminal Procedure Law, probationers are offenders sentenced to limited incarceration or up to 3 years of imprisonment. If the offense is minor, the offender shows remorse and poses no risk of re-offending, and if granting probation is unlikely to significantly harm the community, the court may suspend the sentence for a specified probationary period. Additionally, offenders placed on probation undergo community corrections for the duration of the probation period, managed by correctional institutions in accordance with the law.

In the United States, assessing the risk of future dangerous behaviors is essential in the decision to grant probation; probation is generally deemed appropriate only when this assessment meets a sufficiently low threshold. In other words, evaluating factors associated with future dangerous behaviors has become integral to the probation process in the United States. Research indicates that a key principle of effective probation is to assess each offender’s level of future dangerous behaviors and match correctional strategies accordingly: the most intensive interventions should be assigned to high-risk offenders, while those at lower risk receive less intensive services. This practice has been shown to reduce recidivism (Stevenson, 2018). In addition, integrating the assessment of future dangerous behaviors factors related to with corrections not only helps justice officials reduce the likelihood of recidivism but also enhances community safety. By proactively addressing the criminogenic needs of probationers—particularly those related to future dangerous behaviors—this integrated model aims to facilitate meaningful behavioral change while ensuring community safety (Elijah et al., 2016).

In China, judges consider the assessment of a defendant’s future dangerous behavior when deciding on probation, as they are concerned about the risks of placing the offender under community correction. Due to the uncertainty surrounding future dangerous behaviors and their impact, judges may perceive uncontrollable risks in granting probation. Meanwhile, in China’s corrections practice, numerous studies report that higher assessed risk-of-dangerousness among probationers predicts greater odds of reoffending during community supervision (Zhao, 2017). To be more specific, offenders with a high risk of future dangerous behaviors not only have an extremely high likelihood of recidivism themselves, but also pose a risk of “criminogenic contagion” to other residents in the community (Guan, 2018). Surveys have found that the community corrections institutions face significant challenges in effectively assessing the future dangerous behaviors of probationers, leading to a lack of effective intervention and correction after probation is pronounced (Liu, 2023). From this perspective, it is of great practical significance to improve the quality of the application of the probation system by focusing on and assessing the factors influencing the future dangerous behaviors, facilitating the smooth social integration of probationers and eliminating factors that may lead to recidivism, and helping them to become law-abiding citizens (Guo, 2012).

Surveys in China’s judicial practice show that probationers are more likely to experience social exclusion and deprivation compared to the general population (Lian, 2025). This often leads to a sense of relative deprivation, negatively impacting their physical and mental health (Xiong and Ye, 2016). They may also rationalize past offenses, intensifying the likelihood of violent behavior, drug use, and other criminal acts (Chen et al., 2024). On this basis, some studies have attempted to provide a comprehensive explanatory framework for this mechanism of future dangerous behaviors, trying to integrate other theoretical elements to explore how they work together to influence future dangerous behaviors, such as incorporating the general strain theoretical variable (i.e., negative coping styles) and exploring its mediating role (Xu et al., 2021). Also some researchers have examined the moderating role of the social identity theoretical variable (i.e., self-esteem) (Shreena et al., 2020).

Building on these insights, the present study first examined how relative deprivation affects probationers’ future dangerous behaviors. It then adopts a broader theoretical perspective by integrating additional key constructs—such as negative coping styles and self-esteem—as mediating and moderating variables. Through this integrative approach, we investigated the complex mechanisms linking probationers’ relative deprivation to future dangerous behaviors.

## 2 Literature review and research hypotheses

### 2.1 Relative deprivation and future dangerous behaviors among probationers

Based on surveys from China’s judicial practice, probationers are more prone to reoffend because they compare their situation with others, experience a certain sense of social unfairness, generate a higher sense of relative deprivation, and then experience criminogenic frustration, which increases their level of future dangerous behaviors (Guo, 2012). Relative deprivation refers to an individual’s subjective and negative perception of their own situation, rooted in the psychological process of social comparison. Heather et al. (2012) suggest this perception is not derived from absolute disadvantage but rather from comparison with others. Callan et al. (2015) indicate that relative deprivation negatively affects both individual and collective behavior. It triggers emotions like frustration, stress, and anger, leading individuals to adopt maladaptive coping styles and increasing the likelihood of engaging in illegal and criminal activities.

According to Relative Deprivation Theory (Smith and Yuen, 2014), when individuals compare themselves with a reference group and perceive themselves in an unfavorable situation, they are likely to experience feelings of resentment and anger, which may further lead to the adoption of unconventional or antisocial behaviors. A sense of relative deprivation occurs when individuals perceive that they, or the group to which they belong, is in a disadvantageous position relative to the reference object. For probationers, due to the public disclosure of criminal records information and community corrections, the criminal information and criminal status of probationers are openly spread, which often leads to renewed attention to their past crimes from those around them. This often results in a certain degree of biases against probationers and a heightened sense of relative deprivation when compared to the general population. This comparative disadvantage further strengthens their risk of reoffending (Qiang, 2013). In China’s community correction practice, probationers with a high sense of relative deprivation develop a sense of frustration which naturally provokes their negative emotions, and then release their hostility by violating relevant supervision and management regulations or by committing criminal offenses again (Guo, 2012).

Based on this, the present study proposed Hypothesis 1:

*H1*. *Probationers’ sense of relative deprivation would have a significant increasing effect on their future dangerous behaviors.*

### 2.2 Negative coping styles and future dangerous behaviors among probationers

From the viewpoint of China’s community correctional practice, probationers with high levels of relative deprivation often experience intense negative emotions when coping with the frustration arising from subjective social comparisons. Influenced by these negative emotions, individuals tend to adopt negative coping styles, which in turn increase their future dangerous behaviors (Chai, 2020). Negative coping styles refer to the behavior individuals take when facing stress or dilemma (Jade and Dimity, 2017), which mainly involve adopting negative attitudes such as avoidance or denial to cope with difficulties (Liu, 2023). The General Strain Theory (Agnew, 2001) holds that when an individual experience stress, it generates tension that may lead to various negative emotions which when coped with using 119 negative coping styles increase the probability of the individual committing a criminal act. According to this theory, when individuals face pressure, if they respond in a negative way, there tends to be a great possibility that it will lead to aggression, violent behavior, or even criminal behavior (Du et al., 2023). Ashleigh et al. (2019) indicate that for probationers in the community, if their past experiences have shown that aggressive or violent behaviors successfully resolved problems, they are more likely to resort to these same reactive behaviors in the face of future challenges and frustrations. That is, negative coping styles have a significant effect on the future dangerous behaviors of probationers and increase the probability of reoffending.

Empirical studies suggest that negative coping styles serve as a crucial mediating factor between stressful life events caused by a sense of relative deprivation and aggressive or criminal offenses (Chen et al., 2020). A comparative study in China involving 150 offenders and 160 non-offenders revealed that, under stress, offenders are significantly more inclined to employ negative coping styles and develop a reliance on these styles. This reliance leads them to habitually resort to the same coping mechanisms when confronted with future challenges, which in turn contributes to the recurrence of criminal behaviors (Chai, 2020). According to findings from research about relationship between stress and negative coping styles, it is reasonable to infer that in stressful contexts, individuals with high levels of relative deprivation are more prone to experiencing negative emotions. Influenced by these emotions, they are more likely to adopt negative coping styles, which in turn aggravate their future dangerous behaviors.

Therefore, this research proposed Hypothesis 2:

*H2*. *Negative coping among probationers would mediates the link between relative deprivation and future dangerous behaviors such that higher relative deprivation predicts greater use of negative coping, which in turn predicts higher levels of future dangerous behaviors.*

### 2.3 Self-esteem and future dangerous behaviors among probationers

Previous research has shown that the impact of relative deprivation on future dangerous behaviors among probationers may be mediated by negative coping styles. However, this mediating effect could also be influenced by other variables. To elaborate, not all probationers experiencing relative deprivation adopt negative coping styles, nor do they necessarily exhibit increased future dangerous behaviors. Therefore, it is necessary to examine the moderating role of other variables in this mediating process. Chen et al. (2018) survey from community corrections in China found that probationers with high levels of self-esteem would effectively enhance their subjective awareness and ability to adapt to society and thus reduce the probability of recidivism by expanding their autonomy and rebuilding their subjective social identity as much as possible while serving their community sentences, resulting in positive thinking and increased positive coping styles. Based on these findings, it is essential to investigate the impact of self-esteem on future dangerous behaviors among probationers and determine whether self-esteem acts as a protective moderator in the relationship between relative deprivation, negative coping styles, and future dangerous behaviors.

According to Social Identity Theory, when individuals identify with a particular group and perceive that their personal values align with the core values of the group, they will not only internalize the group’s norms and comply with the rules and regulations, but also positively evaluate and support the group, defending its interests, and actively take on collective responsibilities of the group, which will in turn enhance their self-esteem (Adam, 2020). Based on this, self-esteem is a positive cognitive attitude, it is characterized by high levels of self-efficacy and a strong sense of collective value. As a result, individuals with high self-esteem possess greater psychological resources to cope with adversity, which allows them to effectively regulate the relationship between relative deprivation, negative coping styles, and future dangerous behaviors.

Firstly, individuals with high self-esteem are not only able to accurately position themselves and positively accept their own identity but also possess a strong sense of collective identification. Therefore, when experiencing relative deprivation, such individuals do not allow this negative perception to dominate their behavior. Instead, they positively evaluate themselves and embrace collective values, thereby suppressing negative emotional and behavioral responses and reducing the probability of engaging in criminal activities (Shreena et al., 2020). Secondly, research indicates that individuals with high self-esteem have greater psychological resources to cope with adversity (Ulrich and Richard, 2022). Thus, even when high self-esteem level probationers experience relative deprivation due to social comparisons, they are less likely to resort to negative coping styles. Empirical surveys have found that probationers with high self-esteem, despite experiencing negative emotions stemming from relative deprivation, utilize various opportunities, institutional support, and social networks to expand their sense of empowerment (LeBel, 2012; Bazemore and Erbe, 2004). They actively express their needs and basic rights within the legal framework, communicate with community correction agencies, and integrate into the community. This proactive engagement strengthens their positive interaction with the community, reducing the likelihood of adopting negative coping styles. Thirdly, even if individuals with high self-esteem occasionally exhibit negative coping styles due to relative deprivation, they are more likely to employ self-serving strategies to overcome these challenges. This process inhibits maladaptive behaviors and positively influences their psychological state through internal cognitive evaluation, thereby reducing the probability of reoffending (Tian et al., 2018).

Based on these insights, this research proposed Hypotheses 3, 4, and 5:

*H3*. *The self-esteem of probationers can negatively moderate the relationship between relative deprivation and future dangerous behaviors. The positive effect of relative deprivation on future dangerous behaviors is weaker at higher levels of self-esteem.*

*H4*. *The self-esteem of probationers can negatively moderate the relationship between relative deprivation and negative coping styles. The positive effect of relative deprivation on negative coping is weaker at higher levels of self-esteem.*

*H5*. *The self-esteem of probationers can negatively moderate the relationship between negative coping styles and future dangerous behaviors. The positive effect of negative coping on future dangerous behaviors is weaker at higher levels of self-esteem.*

Based on the previous analyses, the relationships revealed by Hypotheses 1–5 can be further conceptualized as a moderated mediation model. Specifically, as the level of self-esteem among probationers increases, the positive mediating effect of negative coping styles on the relationship between relative deprivation and future dangerous behaviors weakens. Conversely, when self-esteem is lower, the positive mediating effect of negative coping styles on this relationship gets stronger.

Accordingly, this research proposed Hypothesis 6:

*H6*. *The self-esteem of probationers can negatively moderate the positive mediating effect of negative coping styles in the relationship between relative deprivation and future dangerous behaviors, demonstrating itself as a moderated mediating effect. That is, the mediating role of negative coping styles in the relationship between relative deprivation and future dangerous behaviors diminishes as the level of self-esteem increases, and vice versa.*

## 3 Methods

### 3.1 Data

Data for the present research were collected in 2022–2024, using a multi-stage stratified sampling method. The sampling was conducted in three stages based on the hierarchical criteria of “province/municipality—city/district—township/town/sub-district judicial office.” Initially, four provinces representing the eastern (Jiangsu Province), southern (Guangdong Province), western (Yunnan Province), and northern (Beijing Municipality) regions of China were selected. Within each province, 2–3 cities’ justice bureaus were chosen for investigation, totaling 12 cities. In each city, 2 urban judicial offices and 2 suburban judicial offices were selected as survey sites, resulting in a total of 48 judicial offices. Following this procedure, 1,130 probationers were selected from the 48 judicial offices as the survey sample, averaging 23.5 per office. Structured questionnaires were distributed and collected on the spot in the training rooms of the judicial offices under the guidance of professionally trained survey supervisors. A total of 1,085 questionnaires were collected. After excluding samples with incomplete demographic variables, the final effective sample size was 1,023, with an effective questionnaire recovery rate of 90.5%. Considering the missing data, we conduct the Little’s test, result shows: *x*^2^/df = 1.206, *p* > 0.05. The results indicate that the missing data in this study were missing at complete random and the missing values were handled by EM interpolation.

### 3.2 Procedures

Prior to the commencement of the structured questionnaire survey, all probationers included were required to provide their written consent. Meanwhile, the distribution of the questionnaires and data collection were conducted by trained survey supervisors with backgrounds in psychology and sociology. In addition, all participating probationers were informed that they could freely withdraw and terminate the survey at any time. They were also assured that the questionnaires did not require the use of real names, would not be subject to review by community correction departments, and that their responses would be strictly confidential, to make this credible, surveys were completed in private without officers present, contained no identifying fields, and were deposited by participants into a locked box for researcher-only retrieval. The collected data were used solely for academic research, thereby ensuring the voluntariness, anonymity, and confidentiality of the survey.

### 3.3 Measurement of variables

#### 3.3.1 Dependent variable

This study employed the “Future Dangerous Behaviors Questionnaire (FDQ)” to measure the dependent variable “future dangerous behaviors,” aiming to assess participants’ risk of committing new offenses that result in imprisonment or community correction during their probation period or after release (Yu and Zhang, 2004). The questionnaire covers six dimensions: criminal history, crime attribution, drug-related status, undesirable habits, psychological and physiological conditions, and demographic information. It contains 25 items including “residence status before the crime,” “emotional stability before the crime,” “drug use or drug trafficking experience before the crime,” Taking “residence status before the crime” as an example: respondents were assigned a score of 0 if they had a stable residence before the crime; a score of 2 if they had a relatively stable residence; and a score of 5 if they were without a stable residence or experienced long-term homelessness. Higher scores indicate a higher extend for future dangerous behaviors. The Cronbach’s alpha for the FDQ was 0.978.

#### 3.3.2 Independent variables

The independent variables in this study were relative deprivation, negative coping styles, and self-esteem. The following maturity scales were employed as measurement tools: the Relative Deprivation Questionnaire (RDQ), the Coping Style Questionnaire (CSQ), and the Self-Esteem Scale (SES), as detailed below.

*Relative Deprivation Questionnaire (RDQ)*. The RDQ assesses the sense of deprivation that respondents experience when comparing themselves to a relevant reference group (Ma, 2012). This 4-item measure uses a 6-point rating scale, ranging from 1 (strongly disagree) to 6 (strongly agree), with items such as “Given the effort and sacrifices I have made, my life should be better than it is now” and “I always feel that others have taken what rightfully belongs to me.” Higher total scores indicate a stronger sense of relative deprivation. In this study, the Cronbach’s alpha for the RDQ was 0.976.

*Coping Style Questionnaire (CSQ)*. The CSQ mainly evaluates the coping styles respondents employ when facing stress or adversity (Fu et al., 2012). The questionnaire uses a 5-point scale from 1 to 5 and includes 2 sub-questionnaires: positive coping style and negative coping styles. In this research, one of the sub-questionnaires, the negative coping styles, was used, which comprises 10 items, such as “Easily caught up in memories and fantasies of the event and unable to get rid of them,” and “Easily angry with others and often lose temper.” Items are rated on a 5-point scale (1 = definitely not, 5 = definitely yes), with higher scores indicating a greater tendency for subjects to adopt negative coping styles. The Cronbach’s alpha for this questionnaire was 0.989.

*Self-Esteem Scale (SES)*. The SES focuses on the overall cognitive attitudes that participants hold toward the psychology and behavior of the self (Niu et al., 2016). This 9-item measure uses a 4-point rating scale, ranging from 1 (strongly disagree) to 4 (strongly agree), with items such as “I feel that I am a valuable person, at least at the same level as others” and “I have a positive attitude toward myself.” Higher total scores indicate higher levels of self-esteem. The Cronbach’s alpha for the SES in this study was 0.969.

#### 3.3.3 Control variables

In order to avoid any unrelated variables from affecting the relationships between the variables in this study, age, gender, educational background, crime type, sentencing term, and residency status of the probationers were used as control variables because previous research has shown that these variables are related to future dangerous behaviors. Therefore, these factors were measured during participant selection. First, age was controlled, as studies have shown that differences in future dangerous behaviors can be partially explained by age (Christopher, 2000). Second, gender was controlled, as research indicates that future dangerous behaviors differs between males and females (Brent, 2005). Third, education level was controlled as an important indicator of an individual’s knowledge and quality, marking their level of cultural education and development, which is significantly associated with future dangerous behaviors (Brian et al., 2022). Fourth, crime type and sentencing term were controlled, as studies have shown that these factors effectively influence future dangerous behaviors (Daniel et al., 2016; John et al., 2014). Finally, residency status was controlled, as in the Chinese context, non-local registrants, who are at a disadvantage, are more likely to experience relative deprivation and thus have a higher risk of future dangerous behaviors compared to local registrants (Zhang et al., 2022). Therefore, in order to reduce the effects from age, gender, educational background, crime type, sentencing term, and residency status, this study added the above variables as control variables to the regression model in order to control the interference of these factors on the results of the study.

### 3.4 Statistical analyses

The data processing and analysis procedures of our research were as follows: First, confirmatory factor analysis was conducted to test the discriminant validity of the latent variables, and Harman’s single-factor test was used to examine common method bias, in order to assess whether the four variables involved in this study have good discriminant validity and whether there is any common method bias in the data. Second, we described sample characteristics (age, gender, education, crime type, sentence length, residency) and ran zero-order correlations to gauge suitability for path analyses. Although the data were collected hierarchically (individuals within judicial offices and cities), unconditional models indicated near-zero intraclass correlations and design effects <2; therefore, we retained ordinary least squares (OLS) models with heteroskedasticity-robust standard errors clustered at the judicial-office level. Third, based on the descriptive statistical analysis, we conducted correlation analyses of the variables in this study to determine whether they were suitable for further mediation and moderation analyses. Fourth, building on the previous analyses, we tested the mediating effect of negative coping styles between relative deprivation and future dangerous behaviors, and used the bias-corrected percentile bootstrap method to calculate the 95% confidence interval (CI) to verify Hypotheses 1 and 2 of this study. Fifth, we examined the moderating role of self-esteem in the relationships among relative deprivation, negative coping styles, and future dangerous behaviors, in order to test Hypotheses 3, 4, and 5 of this study. Sixth, following the path analysis technique proposed by Wen and Ye, we tested a moderated mediation model to examine Hypothesis 6 of this study. In summary, the simplified roadmap of the six main analytical stages in this study is: 1. CFA → 2. Descriptive → 3. Correlation → 4. Mediation → 5. Moderation → 6. Moderated Mediation.

## 4 Result

### 4.1 Confirmatory factor analysis and common method biases

To assess the discriminant validity of the latent variables among relative deprivation, negative coping styles, self-esteem, and future dangerous behaviors, we conducted a confirmatory factor analysis (CFA). The results of the CFA, as shown in Table 1, indicate that the four-factor model provided the best fit compared to other models (x^2^/df = 2.561, RMSEA = 0.064, CFI = 0.934, TLI = 0.905, SRMR = 0.045). These indices suggest that the four variables examined are well-differentiated constructs, that is, the correspondence between the relative deprivation questionnaire (RDQ), negative coping styles questionnaire (NCSQ), self-esteem scale (SES) and future dangerous behaviors questionnaire (FDQ) adopted in this study and the measurement items is in line with theoretical expectations.

**Table 1:** Confirmatory factor analysis by comparing alternative measurement models.

Model	*χ2*	*df*	*χ2/df*	RMSEA	CFI	TLI	SRMR
Four factor model: RD; NC; SE; FD	2755.56	1,076	2.561	0.064	0.934	0.905	0.045
Three factor model: RD + NC; SE; FD	4071.86	1,077	3.781	0.076	0.840	0.869	0.059
Three factor model: RD + SE; NC; FD	4221.34	1,077	3.919	0.084	0.839	0.858	0.061
Three factor model: RD + FD; NC; SE	4534.67	1,077	4.210	0.087	0.788	0.777	0.065
Three factor model: RD; NC + SE; FD	4985.71	1,077	4.629	0.089	0.741	0.729	0.069
Two factor model: RD + NC + SE; FD	5743.52	1,079	5.323	0.098	0.712	0.699	0.077
One factor model: RD + NC + SE + FD	9321.38	1,080	8.631	0.155	0.682	0.668	0.087

*N* = 1,023. RMSEA, root mean square error of approximation; CFI, comparative fit index; TLI, tacker-lewis index; SRMR, standardized root mean square residual; RD, Relative Deprivation; NC, Negative Coping Styles; SE, Self-Esteem; FD, Future Dangerous Behaviors; “+” represents that two factors are combined into one factor.

To further address potential common method biases (CMB), given that all four variables (relative deprivation, negative coping styles, self-esteem, and future dangerous behaviors) were derived from self-reports of probationers, Harman’s single-factor test was conducted. The exploratory factor analysis of all items related to these variables revealed that the first factor explained 22.7% of the variance, which is below the 40% criterion typically used to indicate the presence of significant CMB. Moreover, the single-factor model exhibited poor fit indices (x^2^/df = 8.631, RMSEA = 0.155, CFI = 0.682, TLI = 0.668, SRMR = 0.087), this indicates that the measurement items of the four variables do not all belong to the same factor, thus suggesting that there is no evidence of significant common method bias in the present study.

### 4.2 Descriptive statistical analysis

The age of the surveyed probationers ranged from 18 to 71 years, with a mean age of 36.12 ± 11.78 years, the median of age was 36 (*P*_25_ = 27, *P*_75_ = 46). The sample included 754 males (73.70%) and 269 females (26.30%). Educational background was distributed as follows: 128 individuals (12.51%) had an elementary school education or lower, 581 (56.79%) had a junior high school education, 186 (18.18%) had a senior high school education, and 128 (12.51%) had a college education or higher. Crime types, based on the classification by Bai (2010), were distributed as follows: 165 individuals (16.13%) committed violent public offenses, 244 (23.85%) committed violent private offenses, 497 (48.58%) committed non-violent public offenses, and 117 (11.44%) committed non-violent private offenses. Sentencing terms were distributed as follows: 435 individuals (42.52%) had sentences of less than 1 year, 359 (35.09%) had sentences of 1–2 years, and 229 (22.39%) had sentences of 2–3 years. Regarding residency status, nearly 70% of the probationers were local residents (*n* = 692, 67.64%). The composition of the sample is shown in Table 2.

**Table 2:** Sample description.

Individual characteristics	Category	Quantity	Percentage
Age	≦25	115	11.24%
	26–35	323	31.57%
	36–45	291	28.45%
	46–55	235	22.97%
	≧56	59	5.77%
Gender	Male	754	73.70%
	Female	269	26.30%
Education background	Primary school and below	128	12.51%
	Secondary school	581	56.79%
	High school	186	18.18%
	College and above	128	12.51%
Crime type	Violent public right crime	165	16.13%
	Violent private right crime	244	23.85%
	Non-violent public right crime	497	48.58%
	Non-violent private right crime	117	11.44%
Sentencing term	≦12 months	435	42.52%
	13–24 months	359	35.09%
	25–36 months	229	22.39%
Residency status	Local status	692	67.64%
	Migration status	331	32.36%

N = 1,023. The tail difference of percentages is adjusted at the end of each item.

### 4.3 Correlation analyses

Pearson’s correlation analyses was conducted on the variables of age, gender, education background, crime type, length of sentence, residency status, relative deprivation, negative coping styles, self-esteem and future dangerous behaviors (see Table 3). The results revealed significant pairwise correlations among relative deprivation, negative coping styles, self-esteem, and future dangerous behaviors. This suggests that individuals who feel more deprived and use negative coping strategies are more likely to report dangerous behavioral tendencies, while those with higher self-esteem are less likely to do so. These results provide preliminary support for the assumptions of the study.

**Table 3:** Correlations among the variables.

Variables	Mean	SD	1	2	3	4	5	6	7	8	9	10
1. Age	2.804	1.090	–									
2. Gender	1.263	0.440	0.026	–								
3. Education Background	2.307	0.714	−0.086^**^	−0.001	–							
4. Crime type	2.553	0.799	0.075^*^	−0.119^***^	0.061	–						
5. Sentencing Term	1.799	0.609	0.025	0.003	0.018	−0.028	–					
6. Residency Status	1.324	0.468	−0.004	0.137^***^	0.070^*^	−0.056	−0.020	–				
7. Relative Deprivation	14.105	7.257	0.153^***^	−0.173^***^	0.012	0.191^***^	0.260^***^	−0.138^***^	–			
8. Negative Coping Styles	30.614	13.360	0.139^***^	−0.161^***^	−0.001	0.163^***^	0.235^***^	−0.094^**^	0.875^***^	–		
9. Self-esteem	22.670	8.237	−0.140^***^	0.211^***^	0.053	−0.195^***^	−0.167^***^	0.123^***^	−0.790^***^	−0.717^***^	–	
10. Future Dangerous Behaviors	74.145	41.887	0.164^***^	−0.157^***^	−0.007	0.168^***^	0.275^***^	−0.131^***^	0.895^***^	0.932^***^	−0.717^***^	–

*N* = 1,023. Age: ≦25 = 1, 26–35 = 2, 36–45 = 3, 46–55 = 4, ≧56 = 5. Gender: male = 1, female = 2. Education Background: primary school and below = 1, secondary school = 2, high school = 3, college and above = 4. Crime Type: violent public right crime = 1, violent private right crime = 2, non-violent public right = 3, non-violent private right = 4. Sentencing Term: ≦12 months = 1, 13–24 months = 2, 25–36 months = 3. Residency Status: local status = 1, migration status = 2. *p < 0.05, **p < 0.01, ***p < 0.001.

### 4.4 Hypothesis testing

In order to test theoretical hypotheses 1–6, we first converted the control variables age, gender, educational background, crime type, length of sentence, and residency status into dummy variables, and then followed MacKinnon’s four-step procedure to test the mediation effect of negative coping styles, and test the moderating effect of self-esteem. Finally, we verify the moderated mediation model. The results are presented in Table 4.

**Table 4:** Results for multi-level regression model.

Variables	Model 1 (criterion: FD)	Model 2 (criterion: NC)	Model 3 (criterion: FD)	Model 4 (criterion: FD)	Model 5 (criterion: NC)	Model 6 (criterion: FD)
Control variables						
Age	0.027	0.004	0.024^*^	0.012	0.009	0.011
Gender	−0.006	−0.015	0.004	0.006	−0.011	0.003
Education background	−0.016	−0.013	−0.008	−0.009	−0.008	−0.013
Crime type	−0.001	−0.004	0.002	−0.010	−0.001	−0.014
Sentencing term	0.047^**^	0.008	0.041^***^	0.038^***^	0.012	0.035^***^
Residency status	−0.007	0.030	−0.026^*^	−0.021^*^	0.027	−0.019^*^
Independent variable						
Relative deprivation	0.877^***^	0.875^***^	0.320^***^	0.620^*^	0.992^***^	0.503^***^
Mediating variable						
Negative coping styles			0.636^***^	0.653^*^		0.057^*^
Moderating variable						
Self-esteem				−0.236^***^	−0.057^**^	−0.361^***^
Interaction						
RD × SE				−0.234^***^	−0.114^**^	−0.116^**^
NC × SE						−0.444^***^
R^2^	0.804	0.767	0.898	0.905	0.771	0.914
Adjusted R^2^	0.802	0.766	0.897	0.904	0.769	0.913
F	593.401^***^	478.385^***^	1112.615^***^	964.993^***^	378.426^***^	974.225^***^

*N* = 1,023. RD, Relative Deprivation; NC, Negative Coping Styles; SE, Self-Esteem; FD, Future Dangerous Behaviors; Beta values are standardized coefficients. *p < 0.05, **p < 0.01, ***p < 0.001.

First, results show that controlling for the variables of age, gender, educational back-ground, type of offense, length of sentence, and residency status, Model 1 showed that relative deprivation was a positive predictor of future dangerous behaviors (*β* = 0.877, *p* < 0.001, 95%CI [4.889, 5.233]), indicating that the relative deprivation of probationers had a contributory effect on future dangerous behaviors, thereby supporting Hypothesis 1. Model 2 revealed that relative deprivation significantly predicted negative coping styles (*β* = 0.875, *p* < 0.001, 95%CI [1.551, 1.670]), indicating that relative deprivation promotes negative coping styles among probationers. Model 3 showed that negative coping styles significantly predicted future dangerous behaviors (*β* = 0.636, *p* < 0.001, 95%CI [1.866, 2.122]), suggesting that negative coping styles exacerbate future dangerous behaviors. Meanwhile, relative deprivation still had a significant direct effect on future dangerous behaviors (*β* = 0.320, *p* < 0.001, 95%CI [1.608, 2.089]), indicating that negative coping styles partially mediate the relationship between relative deprivation and future dangerous behaviors. To further examine the mediating effect of negative coping styles under different levels of self-esteem, the bias-corrected percentile Bootstrap method was employed. A total of 5,000 bootstrap samples were created from the original dataset through random sampling. The results indicated that the indirect effect of negative coping styles was 3.212 (*p* < 0.001), with a 95% confidence interval (CI) of [0.517, 0.598]. The mediation effect accounted for 63.47% of the total effect [3.212 / (3.212 + 1.849)]. Accordingly, Hypothesis 2 was supported. This demonstrates that the relative deprivation among probationers not only had a significant direct impact on future dangerous behaviors, but also exerted a significant influence on future dangerous behaviors through the mediating effect of negative coping styles. That is, the relative deprivation among probationers positively predicted their negative coping styles and indirectly positively affected future dangerous behaviors through these styles.

Second, to test the moderating effect of self-esteem, we employed the method suggested by Muller et al. to test Hypotheses 3, 4, and 5. Model 4 revealed that, after controlling for age, gender, education background, crime type, sentencing term, and residency status, the interaction term between relative deprivation and self-esteem significantly negatively predicted future dangerous behaviors (*β* = −0.234, *p* < 0.001, 95%CI [−0.107, −0.068]). This indicates that self-esteem negatively moderated the effect of relative deprivation on future dangerous behaviors, thereby supporting Hypothesis 3. Additionally, Model 5 showed that the interaction term between relative deprivation and self-esteem significantly negatively predicted negative coping styles (*β* = −0.114, *p* < 0.01, 95%CI [−0.023, −0.004]). This suggests that self-esteem negatively moderated the effect of relative deprivation on negative coping styles, thus validating Hypothesis 4. Moreover, Model 6 found that the interaction term between negative coping styles and self-esteem also significantly negatively predicted future dangerous behaviors (*β* = −0.444, *p* < 0.001, 95%CI [0.065, 0.097]). This finding suggests that self-esteem negatively moderated the effect of negative coping styles on future dangerous behaviors, supporting Hypothesis 5.

Simple slope analysis indicated that higher self-esteem weakened the positive influence of relative deprivation on future dangerous behaviors, that is, for probationers with low self-esteem, relative deprivation had a stronger impact on future dangerous behaviors (*β* = 0.854, t = 34.599, *p* < 0.001). In contrast, for probationers with high self-esteem, the impact of relative deprivation on future dangerous behaviors was not significant (*β* = 0.137, t = 3.460, *p* > 0.05). At the same time, higher level of self-esteem also weakened the positive influence of relative deprivation on negative coping styles, that is, with the improvement of the self-esteem level of probationers, the positive predictive effect of relative deprivation on negative coping styles weakened (from *β* = 0.774, t = 25.835, *p* < 0.001 to *β* = 0.362, t = 7.723, *p* < 0.05). In addition, higher self-esteem also weakened the positive influence of negative coping styles on future dangerous behaviors, that is, with the improvement of the self-esteem level of probationers, the positive predictive effect of negative coping styles on future dangerous behaviors weakened (from *β* = 0.511, t = 16.811, *p* < 0.001 to *β* = 0.175, t = 8.348, *p* < 0.01). This demonstrates that self-esteem functions as a positive cognitive resource, enabling individuals to mobilize more coping capacity in the face of difficulties, it not only negatively moderated the direct predictive effect of the relative deprivation of probationers on future dangerous behaviors, but also negatively moderated the relationship between relative deprivation and negative coping styles, as well as the relationship between negative coping styles and future dangerous behaviors.

Finally, we tested hypothesis 6. Following the model analysis technique proposed by Wen and Ye (2014), we further examined the moderated mediation effect, with detailed results presented in Table 5. When self-esteem was low, negative coping styles played a highly significant mediating role between relative deprivation and future dangerous behaviors (indirect effect = 2.225, 95% CI [0.349, 0.440]). In contrast, when self-esteem was high, the mediating role of negative coping styles between relative deprivation and future dangerous behaviors was no longer significant (indirect effect = 0.255, 95% CI [−0.177, 0.039]). The difference between these two conditions was −1.97, which reached significance (*p* < 0.001). Thus, Hypothesis 6 was supported, this showing that the mediating role of negative coping styles between relative deprivation and future dangerous behaviors was also negatively moderated by self-esteem.

**Table 5:** Conditional indirect effect of relative deprivation on future dangerous behaviors at low (−1 SD), Medium (Mean), and High (+1 SD) levels of self-esteem.

Grouping variables	Indirect effect	*SE*	95%CI(LL, UL)
Low self-esteem	2.225^***^	0.024	0.349, 0.440
Medium self-esteem	1.512^*^	0.034	0.272, 0.401
High self-esteem	0.255	0.021	−0.177, 0.039

N = 1,023. SE, standard error. *p < 0.05, **p < 0.01, ***p < 0.001.

In summary, the moderated mediation model proposed in this study was supported. Specifically, relative deprivation among probationers not only directly and positively predicted future dangerous behaviors but also indirectly predicted it through the mediating role of negative coping styles. Moreover, both the direct effect of relative deprivation on future dangerous behaviors and the mediating effect of negative coping styles were negatively moderated by self-esteem.

## 5 Discussion and limitation

### 5.1 Discussion

First, results from Model 1 show that relative deprivation among probationers positively predicts future dangerous behaviors (Webber, 2022). Wang et al. (2023) report a direct correlation between relative deprivation and aggressive behaviors. Negative emotions resulting from increased relative deprivation can also increase future dangerous behaviors. Peng et al. (2022) found that individuals experiencing high levels of relative deprivation may exhibit impulsive and aggressive behaviors driven by negative emotional responses like fury and frustration, which can lead to irrational decision-making (Xu and Li, 2024). This emotional turmoil is exacerbated by social comparisons that highlight one’s inferior status compared to others, creating a cycle where feelings of inadequacy foster further risky behaviors (Greitemeyer and Sagioglou, 2017). According to General Strain Theory, which posits that social strains, compounded by a sense of relative deprivation, can increase the likelihood of criminal behavior (Aseltine et al., 2000). In conclusion, the evidence suggests that as perceptions of relative deprivation escalate among probationers, their susceptibility to engaging in dangerous or criminal behaviors also increases. The emotional and psychological consequences of feeling deprived act as a catalyst for aggression and may lead to greater involvement in deviant activities as individuals seek to reclaim agency in their lives.

Second, the Model 2 and 3 demonstrate that relative deprivation among probationers significantly predicts negative coping styles, which subsequently intensify future dangerous behaviors. Beshai et al. (2017) found that high levels of deprivation can lead to negative coping strategies, including avoidance and aggression. This evidence indicates that those who feel deprived may resort to negative coping behaviors as a method of dealing with their emotional turmoil (Yang, 2016). Moreover, Lin et al. (2024) highlight that feelings of relative deprivation can escalate negative emotions, such as frustration and anger, ultimately leading to unethical behaviors online. Their findings underscore that the emotional distress induced by experiences of deprivation is a significant precursor to deviant behaviors, linking the emotions experienced by probationers to potential future risks. In addition, negative coping styles can be shaped by relative deprivation. Xu and Li (2024) documented that upward social comparison, which often exacerbates feelings of relative deprivation, can lead to increased social anxiety, ultimately influencing further dangerous behaviors. Therefore, relative deprivation among probationers, negative coping styles likely emerge, which indirectly pave the way for future dangerous behaviors.

Third, self-esteem also played a role in the impact of relative deprivation and negative coping styles on future dangerous behaviors. It not only moderated the direct impact of relative deprivation on future dangerous behaviors but also weakened the relationships of relative deprivation to negative coping styles, negative coping styles to future dangerous behaviors, and the indirect pathway linking both through negative coping. Thus, the results of the present study extend the research perspective and content on the relationship between Social Identity Theory and future dangerous behaviors, providing empirical evidence for a multifaceted examination of the mechanisms influencing future dangerous behaviors among probationers (Carrie and Roshni, 2018). Specifically, probationers experiencing relative deprivation during community corrections, yet possessing high self-esteem, are more inclined to adopt positive identity strategies that help them surmount anger and dissatisfaction, thus decreasing the likelihood of aggression or criminal conduct. Moreover, even when probationers experience negative emotions due to relative deprivation, those with high self-esteem actively engage in community-building efforts to gain social recognition, which activates positive emotions and leads them to employ adaptive coping styles to address their challenges. Likewise, probationers with high self-esteem are more apt to employ self-serving strategies to address negative coping tendencies arising from relative deprivation, favoring adaptive emotional regulation that further lowers their risk of future dangerous behaviors (Chen et al., 2018). In summary, self-esteem significantly mediates the relationship between relative deprivation and negative coping styles, ultimately influencing future dangerous behaviors.

Finally, this study offers practical implications for probation service agencies or judicial officers in conducting community correction work. On the one hand, judicial officers should pay close attention to the mediating role of negative coping styles in the relationship between relative deprivation and future dangerous behaviors when carrying out community correction work. For probationers with a high sense of relative deprivation, timely intervention measures should be taken to encourage the use of more positive coping strategies to address problems, in order to prevent the occurrence of reoffending. At the same time, efforts should be made to change probationers’ existing misconceptions, values, and coping styles by strengthening moral education, mental health education, and legal awareness. While emphasizing education, it is also important to consider individual psychological differences and conduct targeted training in coping strategies and behavioral correction. In addition, it is necessary to strengthen probationers’ ability to positively adapt to society by organizing vocational and technical training programs, enabling them to develop basic self-sufficiency during community supervision. This can enhance their social adaptability, help alleviate their psychological stress, and ultimately reduce the likelihood of deviant or even criminal behavior. On the other hand, judicial officers should also pay attention to the moderating role of self-esteem in the process of community corrections. It is important to emphasize enhancing probationers’ self-esteem by helping them gain positive social recognition, thereby fostering their sense of identity and belonging in community activities. Increasing community interactions between probationers and groups such as correctional officers, judicial social workers, and local residents can help them find their place within the community and boost their self-esteem. This not only facilitates their successful reintegration into society but also contributes positively to the stable development of the community (Wang and Chen, 2024).

### 5.2 Limitations of the study

This study had some limitations that can be addressed in future research. First, the theoretical scope needs further expansion. Since the research was based on the concepts of relative deprivation, General Strain Theory, and Social Identity Theory, the exploration of factors influencing future dangerous behaviors among probationers is limited in content. Future research could incorporate additional criminological theories, such as Social Anomie Theory and Social Control Theory, to further uncover the mechanisms influencing future dangerous behaviors among probationers. Second, this study was cross-sectional. Although the structural model reveals the complex relationships among relative deprivation, negative coping styles, self-esteem, and future dangerous behaviors in probationers, it does not sufficiently establish causal relationships among these variables. Therefore, further longitudinal research is needed to validate the causal links proposed in this study. Third, the data sources were relatively limited, as they are primarily based on self-reported questionnaires from probationers, who may exhibit some degree of social desirability or concealment when responding, potentially leading to bias and affecting the accuracy of the data. Future studies should collect data from multiple sources, including judges, community correction officers, and judicial social workers, to enhance the comprehensiveness and scientific nature of data collection and identify more significant factors influencing future dangerous behaviors among probationers. Fourth, the mechanisms influencing future dangerous behaviors may differ among probationers convicted of different types of crimes. Future research should conduct more detailed analyses by categorizing probationers based on specific crime types (e.g., violent crimes, property crimes, drug-related crimes) and integrating the theory of legal interests in criminal law to explore future dangerous behaviors among probationers. Fifth, although the sampling was hierarchical (individuals within offices and cities), unconditional models in our data indicated near-zero ICCs and design effects < 2, nonetheless, the study may be underpowered to detect very small office/city effects, and we did not estimate random slopes nor include office/city covariates. Finally, the participants in this study were probationers from China, therefore the conclusions drawn from the findings are applicable only within the context of the Chinese legal system. Future research could conduct comparative investigations between probationers in China and those in other countries, in order to explore the factors and underlying mechanisms influencing future dangerous behaviors under different cultural contexts. Such an approach would promote deeper cross-cultural research and analysis (Di, 2020). Although preliminary, it’s empirical analysis of the complex relationships between relative deprivation, negative coping styles, self-esteem, and future dangerous behaviors among probationers provides meaningful insights for academic discussions on probation systems, future dangerous behaviors, and community corrections.

## 6 Conclusion

In conclusion, this study expands our understanding of the psychological predictors of future dangerous behavior among probationers. Through a questionnaire survey analysis of 1,023 probationers within China’s judicial system, the results revealed that relative deprivation significantly positively predicts the future dangerous behaviors among probationers. This supports the premise that individuals’ relative deprivation arising from social comparisons increases the probability of engaging in misconduct and criminal offenses. Furthermore, the mediating role of general strain variables (i.e., negative coping styles) provides a robust explanation for the relationship between relative deprivation and future dangerous behavior. Additionally, social identity variables (i.e., self-esteem) negatively moderate both the direct effect of relative deprivation on future dangerous behavior and the mediating effect of negative coping styles. These findings enrich the theoretical model for assessing future dangerous behaviors among probationers and hold significant value for judicial officials in comprehensively evaluating such future dangerous behavior and implementing corresponding intervention and correction measures.

## Data Availability

The raw data supporting the conclusions of this article will be made available by the authors, without undue reservation.
